# Significance of Four Methionine Sulfoxide Reductases in *Staphylococcus aureus*

**DOI:** 10.1371/journal.pone.0117594

**Published:** 2015-02-13

**Authors:** Vineet K. Singh, Manisha Vaish, Trintje R. Johansson, Kyle R. Baum, Robert P. Ring, Saumya Singh, Sanjay K. Shukla, Jackob Moskovitz

**Affiliations:** 1 Department of Microbiology and Immunology, A.T. Still University of Health Sciences, Kirksville, Missouri, United States of America; 2 Marshfield Clinic Research Foundation, Marshfield, Wisconsin, United States of America; 3 Department of Pharmacology and Toxicology, School of Pharmacy, University of Kansas, Lawrence, Kansas, United States of America; University of Illinois at Chicago College of Medicine, UNITED STATES

## Abstract

*Staphylococcus aureus* is a major human pathogen and emergence of antibiotic resistance in clinical staphylococcal isolates raises concerns about our ability to control these infections. Cell wall-active antibiotics cause elevated synthesis of methionine sulfoxide reductases (Msrs: MsrA1 and MsrB) in *S. aureus*. MsrA and MsrB enzymes reduce *S*-epimers and *R*-epimers of methionine sulfoxide, respectively, that are generated under oxidative stress. In the *S. aureus* chromosome, there are three *msrA* genes (*msrA1*, *msrA2* and *msrA3*) and one *msrB* gene. To understand the precise physiological roles of Msr proteins in *S. aureus*, mutations in *msrA1*, *msrA2* and *msrA3* and *msrB* genes were created by site-directed mutagenesis. These mutants were combined to create a triple *msrA* (*msrA1*, *msrA2* and *msrA3*) and a quadruple *msrAB* (*msrA1*, *msrA2*, *msrA3*, *msrB*) mutant. These mutants were used to determine the roles of Msr proteins in staphylococcal growth, antibiotic resistance, adherence to human lung epithelial cells, pigment production, and survival in mice relative to the wild-type strains. MsrA1-deficient strains were sensitive to oxidative stress conditions, less pigmented and less adherent to human lung epithelial cells, and showed reduced survival in mouse tissues. In contrast, MsrB-deficient strains were resistant to oxidants and were highly pigmented. Lack of MsrA2 and MsrA3 caused no apparent growth defect in *S. aureus*. In complementation experiments with the triple and quadruple mutants, it was MsrA1 and not MsrB that was determined to be critical for adherence and phagocytic resistance of *S. aureus*. Overall, the data suggests that MsrA1 may be an important virulence factor and MsrB probably plays a balancing act to counter the effect of MsrA1 in *S. aureus*.

## Introduction

*Staphylococcus aureus* is an aggressive and versatile pathogen that is responsible for a wide array of diseases ranging from pyogenic skin infections to complicated life-threatening diseases such as bacteremia, central nervous system infections, and endocarditis [1,2,3,4]. Treatment of *S*. *aureus* infections is a great challenge because of the ability of the organism to develop or acquire antibiotic resistance. A widespread use of methicillin and other semi-synthetic penicillins has led to the emergence of methicillin-resistant *S*. *aureus* (MRSA) strains that have become prevalent both in the hospitals and the community throughout the world [5,6]. Infections by MRSA strains cause higher mortality and require longer and more expensive medical care than infections caused by methicillin-sensitive *S*. *aureus* [5].

Host phagocytic cells play key roles in determining the extent of bacterial infections. The phagocytic cells induce a respiratory burst and produce superoxide anion that serves as a precursor to generate additional reactive oxygen species (ROS) such as hydrogen peroxide (H_2_O_2_), hydroxyl radical, singlet oxygen, and hypochlorous acid. These highly reactive species lead to the oxidation of DNA, lipids and proteins. *S*. *aureus* produces antioxidant enzymes such as superoxide dismutases, catalase, alkyl hydroperoxide reductases, etc. to defend itself from the ROS [7]. However, the ROS and other oxidizing conditions still cause damage to cellular macromolecules. The ROS oxidize the sulfur atom of protein-bound methionine residues, resulting in methionine sulfoxide (MetO) that typically leads to loss of protein function. MetO are reduced back to methionine by methionine sulfoxide reductase (Msr) enzymes that restore normal protein functions [8]. Oxidation of methionine results in two diastereomeric forms of MetO, *R*-MetO and *S*-MetO, which are reduced by two different Msr enzymes. MsrB is specific for *R*-MetO whereas MsrA is specific for *S*-MetO [9,10].

Msr proteins have also been shown to contribute to the virulence of bacterial pathogens [11,12,13,14,15]. Absence of Msr enzymes reduces the ability of bacterial cells to adhere to eukaryotic cells that probably impacts colonization of the host [13,14,16,17]. In the absence of the Msr enzymes, the integrity of the bacterial surface proteins is compromised and this deficiency may contribute to the reduced bacterial adherence to eukaryotic cells [13,14,16,17]. In addition, reduced Msr activity impacts bacterial survival within phagocytic cells [12].

In *S*. *aureus* chromosome, there are three *msrA* genes (*msrA1*, *msrA2* and *msrA3*) and one *msrB* gene [18]. The *msrA1* and *msrB* genes are co-transcribed in *S*. *aureus* and their expression is induced specifically in response to cell wall-active antibiotics [19]. The expression of *msrA1/msrB* occurs at much higher levels in *S*. *aureus* relative to the expression levels of *msrA2* or *msrA3* genes [20].

In view of multiple *msrA* and *msrB* genes in *S*. *aureus*; with potential roles in virulence [12,21] and oxidative stress tolerance [18,22], mutations were generated in each of the *msrA* and *msrB* genes. Subsequently, three unique *msr* mutants were constructed by combining the individual mutants that included an *msrB* mutant (lacks ability to reduce *R*-MetO), a triple *msrA* mutant (*msrA1*, *msrA2*, *msrA3*; lacks ability to reduce *S*-MetO), and a quadruple *msrAB* mutant (*msrA1*, *msrA2*, *msrA3*, *msrB*; lacks ability to reduce either *R*- or *S*-MetO). These mutants were used to determine the precise roles of Msr proteins in survival of *S*. *aureus* under a variety of stress conditions. The presented data suggest that MsrA2 and MsrA3 play little or no role in staphylococcal protection from oxidative stress or in mice. However, the role of the *msrA1/msrB* locus is complex. While lack of MsrA1 increases the sensitivity of *S*. *aureus* to oxidative stress and host immune defense, the lack of MsrB, to some extent, is actually beneficial to the bacterial organism under these conditions.

## Materials and Methods

### Ethics statement

Animal studies were approved by the A.T. Still University-Kirksville College of Osteopathic Medicine’s Animal Care and Use Committee (IACUC protocol # 166).

### Bacterial strains, plasmids, antibiotics and growth conditions

The bacterial strains and plasmids used in this study are shown in Table 1. *S*. *aureus* cells were grown in tryptic soy broth (TSB) or tryptic soy agar (TSA) and *Escherichia coli* cells were grown in Luria-Bertani broth or Luria-Bertani agar. Plasmids in *E*. *coli* cells were maintained by adding ampicillin at 100 μg ml^-1^, kanamycin at 20 μg ml^-1^, erythromycin at 15 μg ml^-1^ and tetracyclin at 10 μg ml^-1^, when required. *S*. *aureus* mutant strains were cultured with kanamycin at 100 μg ml^-1^, erythromycin at 15 μg ml^-1^ and tetracyclin at 10 μg ml^-1^, when required.

**Table 1:** Bacterial strains used in this study.

Strains	Characteristics	Reference
*S*. *aureus* RN4220	A restriction minus derivative of *S*. *aureus* strain 8325–4	[54]
SH1000	*S*. *aureus* strain 8325–4 with functional RsbU	[25]
SH1000:*msrA1*	SH1000 with mutation in the *msrA1* gene (Kan^R^)	This study
SH1000:*msrA2*	SH1000 with mutation in the *msrA2* gene (Tet^R^)	This study
SH1000:*msrA3*	SH1000 with mutation in the *msrA3* gene (Erm^R^)	This study
SH1000:*msrA1*-*B*	SH1000 with mutation in the *msrA1-msrB* genes (Kan^R^)	This study
SH1000:*msrB*	SH1000 with mutation in the *msrB* gene (Kan^R^)	This study
SH1000:*msrA*	SH1000 with mutation in the *msrA1*, *msrA2* and *msrA3* genes (Kan^R^, Tet^R^, Erm^R^)	This study
SH1000:*msrAB*	SH1000 with mutation in the *msrA1*, *msrA2*, *msrA3*, and *msrB* genes (Kan^R^, Tet^R^, Erm^R^)	This study
BB270	A homogeneous methicillin resistant *S*. *aureus*	[26]
BB270:*msrA1*	BB270 with mutation in the *msrA1* gene (Kan^R^)	This study
BB270:*msrA2*	BB270 with mutation in the *msrA2* gene (Tet^R^)	This study
BB270:*msrA3*	BB270 with mutation in the *msrA3* gene (Erm^R^)	This study
BB270:*msrA1*-*B*	BB270 with mutation in the *msrA1-msrB* genes (Kan^R^)	This study
BB270:*msrB*	BB270 with mutation in the *msrB* gene (Kan^R^)	This study
BB270:*msrA*	BB270 with mutation in the *msrA1*, *msrA2* and *msrA3* genes (Kan^R^, Tet^R^, Erm^R^)	This study
BB270:*msrAB*	BB270 with mutation in the *msrA1*, *msrA2*, *msrA3*, and *msrB* genes (Kan^R^, Tet^R^, Erm^R^)	This study
SH1000+pCU1	SH1000 with plasmid pCU1 (Cam^R^)	This study
SH1000:*msrA*+pCU1	SH1000:*msrA* with plasmid pCU1 (Kan^R^, Tet^R^, Erm^R^, Cam^R^)	This study
SH1000:*msrAB*+pCU1	SH1000:*msrAB* with pCU1 (Kan^R^, Tet^R^, Erm^R^, Cam^R^)	This study
SH1000:*msrA*+*msrA1*	SH1000:*msrA* with pCU1-*msrA1P*-*msrA1* (Kan^R^, Tet^R^, Erm^R^, Cam^R^)	This study
SH1000:*msrAB*+*msrA1*	SH1000:*msrAB* with pCU1-*msrA1P*-*msrA1* (Kan^R^, Tet^R^, Erm^R^, Cam^R^)	This study
SH1000:*msrAB*+*msrB*	SH1000:*msrAB* with pCU1-*msrA1P*-*msrB* (Kan^R^, Tet^R^, Erm^R^, Cam^R^)	This study

Erm^R^, erythromycin resistant; Kan^R^, kanamycin resistant; Tet^R^, tetracycline resistant; Cam^R^, chloramphenicol resistant

### DNA manipulations

Plasmid DNA was isolated using the Qiaprep Miniprep kit (Qiagen Inc). Chromosomal DNA was isolated using a DNAzol kit (Molecular Research Center) from lysostaphin-treated *S*. *aureus* cells according to the manufacturer’s instructions. All restriction and modification enzymes were purchased from Promega. PCR was performed using a Peltier Thermal Cycler-200 system (MJ research). DNA manipulations were carried out using standard procedures. Oligonucleotide primers (Table 2) were obtained from Eurofins.

**Table 2:** Oligonucleotide primers used in this study.

Oligo	Sequence (5’→3’)
P1	ATCAATTACCTTGGCACCTACC
P2	GGATCCTGACTTGATGCCTGGATATG
P3	GGATCCAACTGAAGGAGAAGTTGTG
P4	AAGCTTGGTCTTGATTGCTTGTTGC
P5	GGATCCTGACACATTCAGCATAACCA
P6	AAGCTTCAGATGCACATTCATGTGA
P7	GCTGCTTACAAACATTTCGA
P8	GGATCCGAACGACGTAAAGACAGAGA
P9	GCTAACGTCATTGAATATG
P10	GGAAGTAACCTCTGGATCA
P11	ATCGTACTAAGGTCTAATG
P12	CTTGGTGATAGTCTTCGGCT
P13	ATGGTAGTTGTTTATGTAG
P14	CTCCTCTGAAAATCACTTGT
P15	GTTACACAAGAAAACGGCA
P16	TCATCATCGTGTTTTGGG
P17	AGGATGTTTCTGGTGCATGG
P18	GACACAACTTCTCCTTCAGT

### Construction of *msr* mutants in *S*. *aureus*

Construction of the *msrA1* [22], *msrA2* [18], and *msrB* [23] mutants has been described previously.

To construct a mutation in *msrA1* and *msrB* genes simultaneously, flanking regions (left of *msrA1* and right of *msrB*) were PCR amplified and ligated. Briefly, primer pairs P1 and P2 were used to amplify a 1449 bp DNA fragment (starting 1364 nt upstream of the *msrA1* start codon and going downstream). Another set of primers P3 and P4 were used to amplify an 841 bp DNA fragment (starting 156 nt downstream of the *msrB* stop codon and going further downstream). These two fragments were ligated in vector pTZ18R [24] which simultaneously engineered a unique *Bam*HI site between the ligated fragments to which a 1.7 kb kanamycin-resistance cassette was cloned. This fragment was used to construct a deletion mutant (*msrA1*-*msrB*) in *S*. *aureus* utilizing the methodology described previously for the construction of individual *msrA1* and *msrA2* mutants [18,22].

To construct an *msrA3* mutant, primers P5 and P6 were used to amplify a 1084 bp DNA fragment upstream of *msrA3* (containing 151 nt of the 5’-end of the *msrA3* gene and going upstream). Another set of primers, P7 and P8, were used to amplify a 1047 bp *msrA3* downstream fragment (containing 153 nt of the 3’-end of the *msrA3* gene and going downstream). These two fragments were ligated together in vector pTZ18R to generate a unique *Bam*HI restriction site between the fragments (lacking a significant portion of the *msrA3* gene, from nucleotide position 152–321 with respect to *msrA3* start codon) to which a 1.4 kb erythromycin-resistance cassette was cloned. The above construct was used as a suicidal plasmid to construct a mutation in the *msrA3* gene utilizing a method described previously [18,22].

For *in vitro* and *in vivo* studies, the *S*. *aureus* strain SH1000 [25], which is a *sigB* positive derivative of the *S*. *aureus* strain RN450, was used. Since most MRSA strains are naturally resistant to tetracycline and or erythromycin, a *S*. *aureus* MRSA strain BB270 [26] (sensitive to kanamycin, erythromycin and tetracycline) was used to combine *msr* mutations for antibiotic resistance studies. The individual *msr* mutants were combined in these two *S*. *aureus* strains to generate a triple (mutant of *msrA1*, *msrA2*, and *msrA3* genes) and a quadruple mutant (mutant of *msrA1*, *msrA2*, *msrA3*, and *msrB*).

### Determination of Msr activity

Msr activity in the cell free extract of the wild-type and the *msr* mutants of *S*. *aureus* was determined using 200 μM of Dabsyl-MetO and 20 mM DTT in 50 mM Tris-HCl (pH 7.5) and incubation at 37°C for 30 min, as previously described [18].

### Growth kinetics of the wild-type *S*. *aureus* and its isogenic *msr* mutant under stress

Mid-exponential phase cultures (OD_600_ = 0.6) were diluted 50-fold in a nephelo culture flask (Wheaton) containing 50 ml fresh TSB with a flask-to-medium volume ratio of 6:1. Oxidative and antibiotic stress conditions were imposed by the addition of H_2_O_2_ and oxacillin in TSB to appropriate concentrations. Bacterial growth was subsequently monitored by incubating the flask in a shaking incubator (250 rpm) and measuring turbidity of the liquid culture.

### Determination of the sensitivity of *msrA* mutants to oxidants and cell wall inhibitors

The minimum inhibitory concentrations (MICs) for the wild-type and different *msr* mutant strains of *S*. *aureus* were determined as previously described [27,28]. In addition to H_2_O_2_, the following oxidizing agents were used in MIC determination studies: cumene hydroperoxide that acts as an intracellular source of reactive oxygen species [29], N-ethylmaleimide that oxidizes thiols and increases disulfide bonds in proteins [30], sodium nitroprusside that serves as a nitric oxide donor [31]; methyl viologen (paraquat) that generates superoxide [31].

### Determination of staphyloxanthin production in *msr* mutants

*S*. *aureus* wild-type and its isogenic *msr* mutant strains were grown at 37˚C for 18 h in TSB. Cells were harvested and washed twice with sterile water and the levels of staphyloxanthin in these cells were quantified as described previously [28,32].

### Phagocytic killing of *S*. *aureus msr* mutant

The promyelocytic HL-60 cells (obtained from American Type Culture Collection) were grown in Iscove’s Modified Dulbecco’s Medium (IMDM) (ATCC) with 10% fetal bovine serum (Fisher) and treated with 1.3% dimethyl sulfoxide (Fisher) for 5 days to induce their differentiation into neutrophil-like cells. The differentiated neutrophils were used for phagocytic killing using a method described previously [33,34]. In brief, the neutrophils (1X10^6^ cells) were added with *S*. *aureus* cells (2.5X10^6^ CFUs) (MOI 1:2.5) in a 24-well plate. The plate was centrifuged at 4000 rpm for 10 min and incubated in a CO_2_ incubator at 37˚C for 1 h. The supernatant was gently aspirated and the neutrophils were lysed by the addition of IMDM containing 0.025% Titron X-100. The number of surviving bacteria was enumerated by making serial dilutions and plating of this lysate on TSA plate.

### Adherence of *msr* mutant to A549 lung epithelial cells

Adherence of *S*. *aureus* SH1000 strain and its isogenic *msr* mutants was determined by infection of lung epithelial cells as described previously [35,36]. In these experiments, a mixture of *msr* mutant and wild-type (60:40 ratio) *S*. *aureus* was used to infect the monolayers of A549 cells. The ratio of the mutants cells adhered to the A549 cells after 1 h was enumerated and compared to the ratio of the mutants in the mixture used in these adherence assays.

### Complementation of triple and quadruple mutants

For complementation studies, the triple mutant was complemented *in trans* with *msrA1* and the quadruple mutant was complemented *in trans* with either *msrA1* or *msrB* gene. The *msrA1* and *msrB* coding regions were cloned immediately downstream of a previously described construct, pCU1-*msrA1*P [18]. The resulting constructs pCU1-*msrA1P*-*msrA1* or pCU1-*msrA1P*-*msrB* was transferred into *S*. *aureus* RN4220 by electroporation and subsequently transduced into the triple or quadruple mutants. For comparative studies, wild-type SH1000, and the triple and quadruple mutants were also transformed with the empty plasmid pCU1 [37].

### Levels of Protein A in *S*. *aureus* cells

Total protein was extracted from lysostaphin treated *S*. *aureus* cells, separated by SDS-PAGE, and transferred to a nitrocellulose membrane. The membrane was blocked with 5% skimmed milk and incubated with rabbit antibodies conjugated to horseradish peroxidase (Bio-rad). The membrane was visualized for Protein A using an Opti-4CN substrate kit (Bio-Rad).

### Hemolysis by *msr* deficient *S*. *aureus*

To visualize the hemolysis, 5.0 μl of the overnight cultures of the wild-type *S*. *aureus* SH1000 and the *msr* mutants were spotted on TSA plates with 5% sheep blood agar and the plate was incubated at 37°C for 48 h.

### Survival of wild-type and *msr* mutants in a murine systemic infection model

Wild-type and *msr* mutants were mixed together and then tested in a murine systemic infection model to determine if these mutations had an effect on the ability of the organism to survive *in vivo* as described previously [28,36]. A 0.5 ml mixture of the wild-type and *msr* mutant cells (~1X10^8^ CFU, approximately 40:60 ratio of the wild-type and mutant) was injected into the peritoneal cavity of Swiss white Hla (ICR)CVF female mice (16–20 g) (Hilltop Lab Animals, Inc.) and the fraction of mutants surviving in the spleen and liver of infected mice was enumerated relative to wild-type *S*. *aureus* as described previously [28,36].

### Localization of *S*. *aureus* MsrA1 and MsrB

To determine the localization of MsrA1 and MsrB in *S*. *aureus*, wild type *S*. *aureus* SH1000 culture was grown in TSB to an OD_600_ = 0.3 and treated with 1.2 μg ml^-1^ oxacillin for 2.5 h to induce the synthesis of these proteins as described previously [19,22]. Bacterial cells were harvested and the cytosolic and the cytoplasmic membrane fractions were prepared as described previously [38], separated by 15% SDS-PAGE and subjected to western blot analysis for the presence of MsrA1 and MsrB.

### Statistical analysis

Data were analyzed with a paired *t*-test using a statistical analysis computer program (R for Windows, version 3.0.2, The R Foundation for Statistical Computing). Statistical significance was set at *p*≤.05.

## Results

### Construction of the *msr* mutants

**Fig 1:**
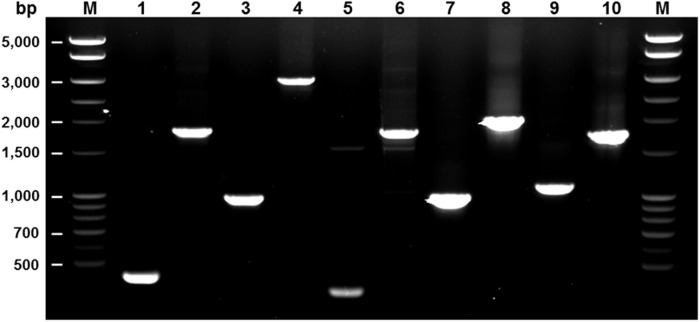
Confirmation of mutations in *msr* genes. Primers from the regions flanking the site of the antibiotic-resistance cassette were used in the PCR. A larger PCR product was observed when genomic DNA from the mutant (even-numbered lanes) was used compared to when wild-type genomic DNA was used (odd-numbered lanes) as a template because of the insertion of a larger antibiotic-resistance cassette. Primers P9 and P10 were used to verify mutation in *msrA1* (Lanes 1 & 2), P11 and P12 for mutation in *msrA2* (Lanes 3 & 4), P13 and P14 for mutation in *msrA3* (Lanes 5 & 6), P15 and P16 for mutation in *msrB* (Lanes 7 & 8), and P17 and P18 for mutation in *msrA1*-*B* (Lanes 9 & 10). Lane M—DNA ladder.

We previously reported the construction and findings of the *msrA1*, *msrA2*, and *msrB* mutants where the phenotypes of the mutant strains were restored by complementation of the mutated genes *in trans* [18,22,23]. The *msrA1* and *msrB* genes in *S*. *aureus* are the first and second of a four-gene operon [18,22]. Also, the *msrA1* mutant produced a significantly higher level of MsrB relative to wild-type *S*. *aureus* [18]. In this study, a mutant was created where the entire *msrA1* and *msrB* gene segments were deleted from the bacterial chromosome and replaced with a kanamycin-resistance cassette to generate an *msrA1*-*msrB* null mutant. An *msrA3* deletion mutant was also constructed. Subsequently, the three *msrA* (*msrA1*, *msrA2*, *msrA3*) mutants were combined to generate an MsrA-deficient triple mutant. In addition, the *msrA2* and *msrA3* individual mutants were combined with an *msrA1-msrB* mutant to generate a quadruple mutant. These mutations were verified by PCR using primer pairs flanking the region that had been deleted in the mutants and replaced by larger antibiotic resistance cassettes (Fig. 1).

### Msr activity in wild-type and *msr* mutants of *S*. *aureus*

Cell-free protein extracts from the wild-type and *msr* mutant cultures were used to determine Msr activity using dabsyl-MetO as a substrate. The Msr activity in various mutants was normalized against the enzymatic activity in the wild-type *S*. *aureus* SH1000 and the data are shown in Table 3. The data demonstrate that the MsrA2 and MsrA3 contribute little to the enzymatic activity in *S*. *aureus* cells (Table 3). An increase in Msr activity in the *msrA1* mutant is because of a higher production of MsrB in this mutant [18]. Further, MsrB is responsible for most of the enzymatic activity (~83%) in wild-type *S*. *aureus* SH1000 (Table 3). There was no enzymatic activity noted in the quadruple *msrAB* mutant (Table 3).

**Table 3:** Methionine sulfoxide reductase activity levels in different *msr* mutants relative to wild-type *S*. *aureus* strain SH1000.

Strain	Percent total activity
Wild-type SH1000	100
SH1000:*msrA1*	218
SH1000:*msrA2*	106
SH1000:*msrA3*	93
SH1000:*msrB*	17
SH1000:*msrA1-B*	19
SH1000:*msrA*	123
SH1000:*msrAB*	0

### Oxidative and antibiotic stress tolerance of the *msr* mutants

**Fig 2:**
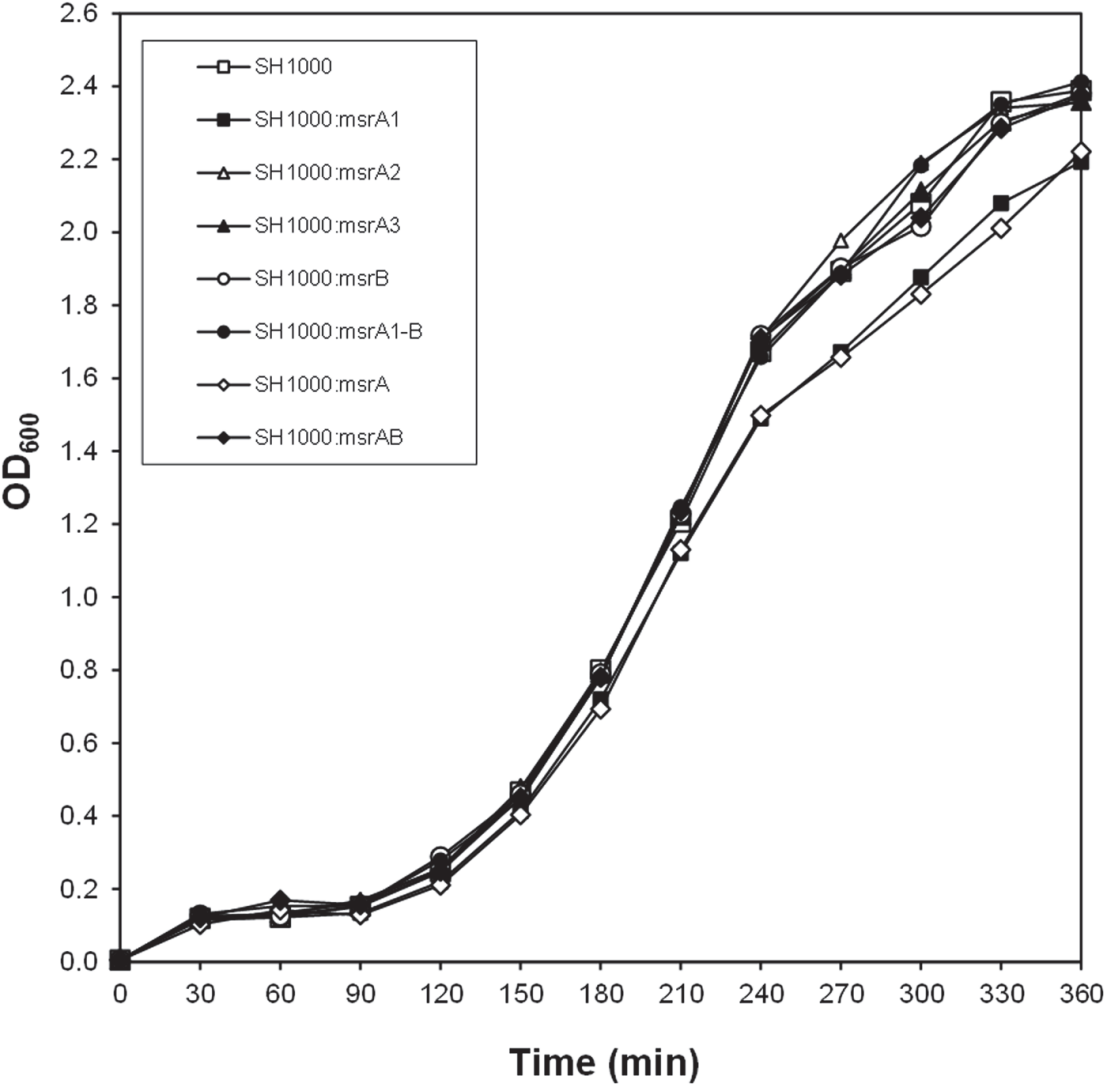
Growth curve of the wild-type *S*. *aureus* strain and its derivative *msr* mutants in TSB. Values indicate the average of two independent experiments.

**Fig 3:**
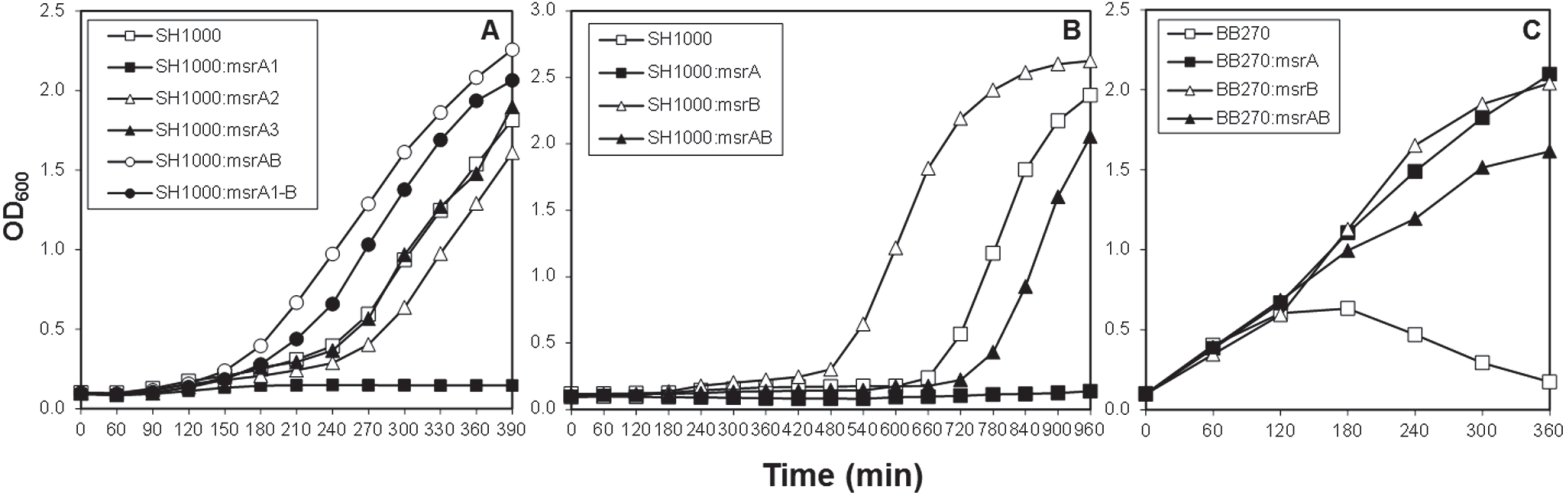
Growth of the wild-type *S*. *aureus* strain and its derivative *msr* mutants in the presence of H_2_O_2_ or oxacillin. (A) growth in the presence of 4.4 mM H_2_O_2_, (B) growth in the presence of 8.8 mM H_2_O_2_ (C) growth in the presence of 400 μg ml^-1^ of oxacillin. Values indicate the average of two independent experiments.

In growth kinetic experiments, the mutants specifically lacking MsrA1 or all three MsrA proteins showed slightly slower growth rate in TSB at 37°C (Fig. 2). Deletion of *msrA2*, *msrA3*, *msrB*, or *msrA1-msrB* had no apparent effect on the growth of the mutant cell compared to the growth of the wild-type *S*. *aureus* SH1000 (Fig. 2). When the Msr deficient mutants were cultured in TSB supplemented with 4.4 mM H_2_O_2_, the *S*. *aureus* strains lacking MsrA1 failed to grow (Fig. 3A). In the case of the combinatorial mutants, no growth was recorded for the triple *msrA* mutant even after 16 h in TSB with 8.8 mM H_2_O_2_ (Fig. 3B). The amount of H_2_O_2_ was raised to 8.8 mM in growth studies utilizing the combinatorial mutants to assess the resistance of MsrB-deficient *S*. *aureus* relative to other strains (Fig. 3B). The *S*. *aureus* strains that lacked MsrB (*msrB*, *msrA1*-*msrB* and the quadruple *msrAB* mutants) were moderately resistant to the presence of H_2_O_2_ in these growth experiments (Fig. 3B). The MsrB-deficient strains of methicillin-resistant *S*. *aureus* BB270 demonstrated better growth even in the presence of a cell wall-active antibiotic, oxacillin (Fig. 3C). In the MIC studies, the *S*. *aureus* strains deficient in MsrB were more resistant to H_2_O_2_ (Table 4). A similar increase in resistance to oxacillin and other cell wall-active antibiotics was observed in the case of MsrB-deficient *S*. *aureus* (Table 5). The strains that lacked MsrA1 were susceptible to oxidative stress conditions and the *S*. *aureus* strain that lacked all three MsrA proteins (the triple *msrA* mutant) showed most sensitivity to oxidants (Table 4). No such increase in sensitivity was noted in MsrA-deficient *S*. *aureus* to cell-wall active antibiotics (Table 5).

**Table 4:** Susceptibilities of *S*. *aureus* parental strain SH1000 and its derivative *msr* mutants to oxidants. MIC values indicate average mM concentrations of three independent experiments.

Strains	H_2_O_2_	CHPO	NEM	SNP	Paraquat
Wild-type SH1000	1	9.5	0.625	250	125
SH1000:*msrA1*	0.5	4.75	0.313	7.81	125
SH1000:*msrA2*	1	9.5	0.625	250	125
SH1000:*msrA3*	1	9.5	0.625	250	125
SH1000:*msrB*	2	9.5	0.625	250	125
SH1000:*msrA1-B*	0.5	9.5	0.625	125	125
SH1000:*msrA*	0.25	2.38	0.313	1.95	31.25
SH1000:*msrAB*	0.5	9.5	0.625	250	125

Abbreviations: H_2_O_2_, hydrogen peroxide; CHPO, cumene hydroperoxide; NEM, N-ethylmaleimide; SNP, sodium nitroprusside.

**Table 5:** Susceptibilities of *S*. *aureus* BB270 parental and *msr* mutant strains to cell wall-active antibiotics.

Strains	Oxacillin	D-cycloserine	Bacitracin
Wild-type BB270	200	75	50
BB270:*msrA1*	200	75	50
BB270:*msrA2*	200	75	50
BB270:*msrA3*	200	75	50
BB270:*msrB*	400	150	100
BB270:*msrA1-B*	300	100	75
BB270:*msrA*	200	75	50
BB270:*msrAB*	200	75	50

MIC values (μg ml^-1^) indicate average of three independent experiments.

### Production of staphyloxanthin pigment in *msr* mutants

**Fig 4:**
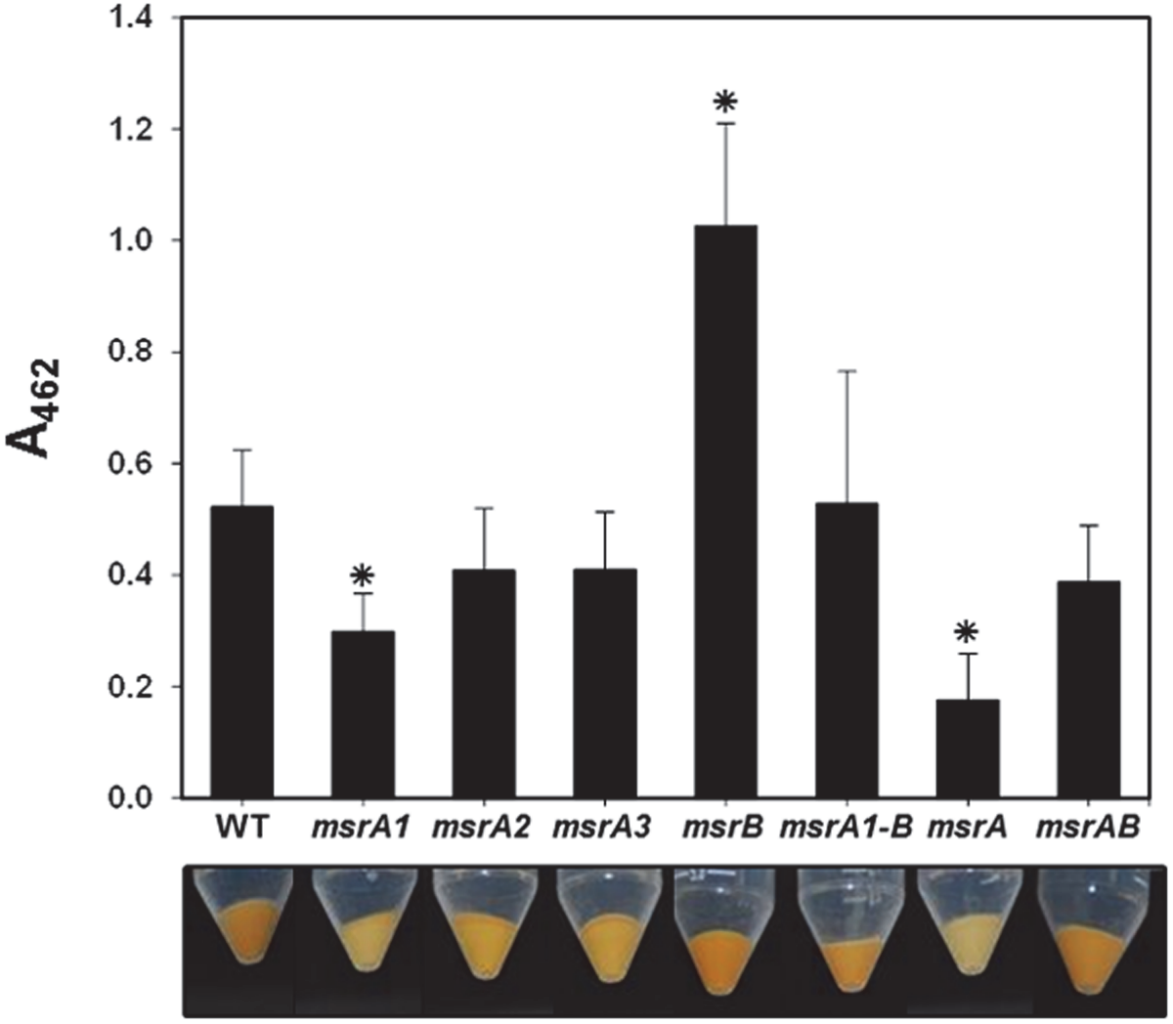
Production of staphyloxanthin in the wild-type *S*. *aureus* strain SH1000 and its derivative *msr* mutants. -The bottom panel shows the color of the bacterial cell pellet from 50 ml overnight grown cultures. The amount of the staphyloxanthin pigment produced by these cells was quantified and is shown as A_462_. Values indicate the average of three independent experiments ± standard deviation (* significant at *p*≤.05).

Of the seven *msr* mutants used in this study, production of staphyloxanthin pigment was highest in the *msrB* mutant strain (Fig. 3). The level of staphyloxanthin was lower in MsrA1-deficient strains (Fig. 3). The MsrA1-deficient *S*. *aureus* has been shown to produce a much higher level of MsrB [18]. Increased pigmentation in MsrB-deficient *S*. *aureus* and reduced pigmentation in cells producing a higher level of MsrB suggests that the MsrB protein suppresses the production of staphyloxanthin in *S*. *aureus*. Production of staphyloxanthin in *msrA2* and *msrA3* mutants was not affected relative to wild-type *S*. *aureus* (Fig. 4).

### Phagocytic killing of the *S*. *aureus msr* mutant cells

**Fig 5:**
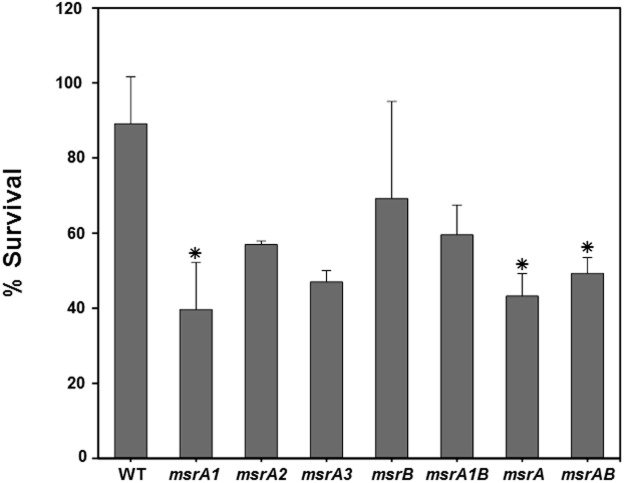
Survival of the wild-type *S*. *aureus* strain SH1000 and its derivative *msr* mutant cells exposed to polymorphonuclear (PMN) cells. PMN cells were infected (MOI 1:2.5) with wild-type *S*. *aureus* SH1000 and its isogenic *msr* mutants for 1 h at 37˚C and then plated on TSA for enumeration. Values indicate the average of three independent experiments ± standard deviation (* significant at *p*≤.05).

Polymorphonuclear cells utilize oxygen-dependent bactericidal pathways in the phagolysosomes. The impact of Msr deletion was investigated on staphylococcal survival in differentiated polymorphonuclear cells. In these studies, the *S*. *aureus* strains with a non-functional MsrA1 showed increased susceptibility to the polymorphonuclear cells (Fig. 5). The survival of the *msrA2*, *msrA3*, *msrB* or *msrA1-B* mutants of *S*. *aureus* was comparable to the wild-type *S*. *aureus* SH1000 in these assays (Fig. 5).

### Role of Msr proteins in adherence of *S*. *aureus* to lung epithelial cells

**Fig 6:**
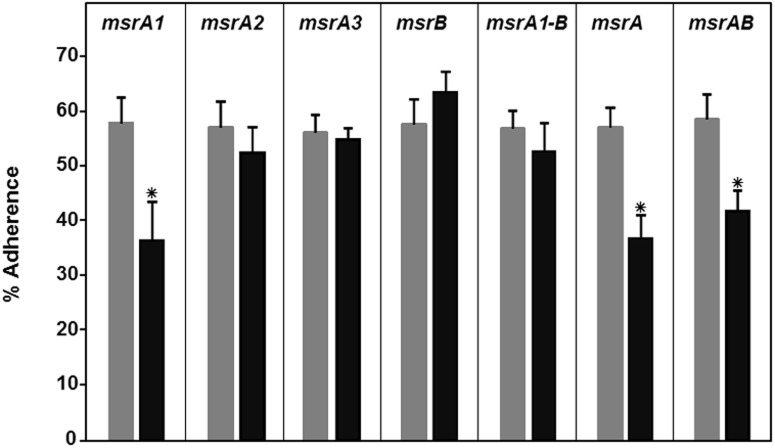
Adherence of the wild-type *S*. *aureus* strain SH1000 and its derivative *msr* mutant cells to A549 human lung epithelial cells. A total of 5X10^5^ bacterial cells were used in these assays. The left light bar in each panel represents the ratios of the *msr* mutant relative to wild-type SH1000 in the mixture used to infect the A549 cells. The right dark bar in each panel represents the ratios of the *msr* mutant in the mixture that adhered to the A549 cells after 1 h of incubation. Values indicate the average of three independent experiments ± standard deviation (* significant at *p*≤.05).

The mixture that was used in adherence assays was biased for an *msr* mutant (~60%) relative to the wild-type *S*. *aureus* SH1000 (~40%). In experiments investigating the adherence of this mixture to A549 cells, the MsrA1-deficient mutants (*msrA1*, *msrA* and *msrAB*) showed significantly reduced adherence (Fig. 6). Deficiency of MsrA2, MsrA3, or MsrB did not impact the adherence of the *S*. *aureus* cells to A549 cells (Fig. 6).

### Protein A levels in *msr* mutants

**Fig 7:**
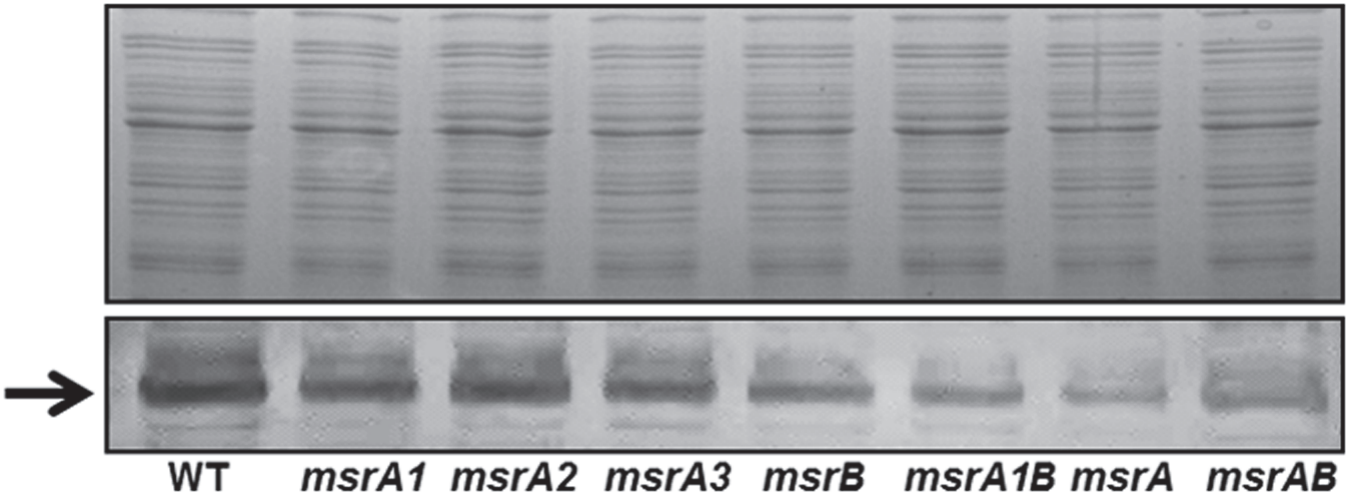
Western analysis of the levels of staphylococcal Protein A in wild-type *S*. *aureus* strain SH1000 and its derivative *msr* mutants. Top panel shows the total protein profile of wild-type and the *msr* mutant strains after SDS-PAGE suggesting that similar amounts of protein were used in the analysis of Protein A. The bottom panel shows the reactivity of Protein A (arrow) in each lane.

Staphylococcal surface protein, Protein A, contributes to bacterial adhesion, virulence, and biofilm formation. In Western blot analysis involving total protein extract from wild-type *S*. *aureus* SH1000 and the derivative *msr* mutant cells, an apparent 55 kDa protein specific to Protein A was detected (Fig. 7). Individual *msr* gene deletions had no appreciable impact on the levels of Protein A in *S*. *aureus* (Fig. 7). However, the protein A-specific band was significantly lighter in the lane corresponding to the triple *msrA* mutant (Fig. 7, Lane 7).

### Hemolytic pattern of *msr* mutants

**Fig 8:**
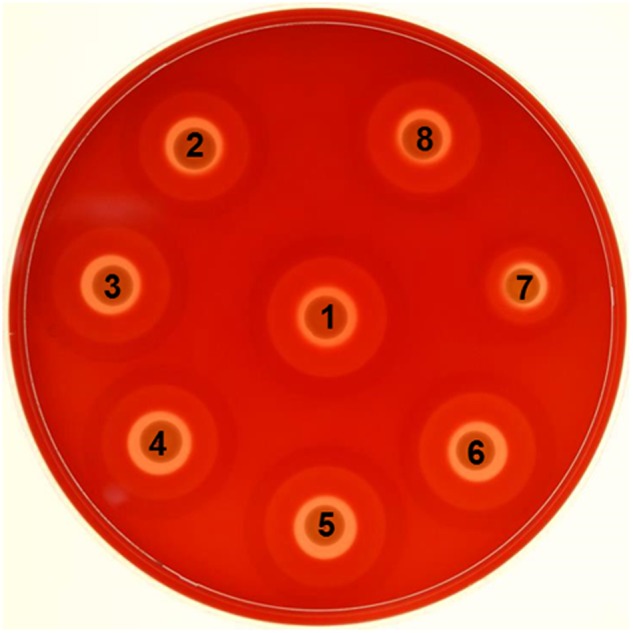
Hemolytic pattern of the wild-type *S*. *aureus* strain SH1000 and its derivative *msr* mutants after 48 h of growth on 5% sheep blood agar plates. (1) Wild-type SH1000, (2) SH1000:*msrA1*, (3) SH1000:*msrA2*, (4) SH1000:*msrA3*, (5) SH1000:*msrB*, (6) SH1000:*msrA1*-*B*, (7) SH1000:*msrA*, (8) SH1000:*msrAB*.

In qualitative assays, the *S*. *aureus* strain that lacked all three MsrA proteins showed a relatively smaller zone of beta-hemolysis relative to other strains (Fig. 8, Spot 7). Another interesting observation was the presence of a significantly reduced secondary zone of hemolysis for the triple mutant (Fig. 8, Spot 7).

### Hemolysis, phagocytic survival and adherence of complemented triple and quadruple mutants

**Fig 9:**
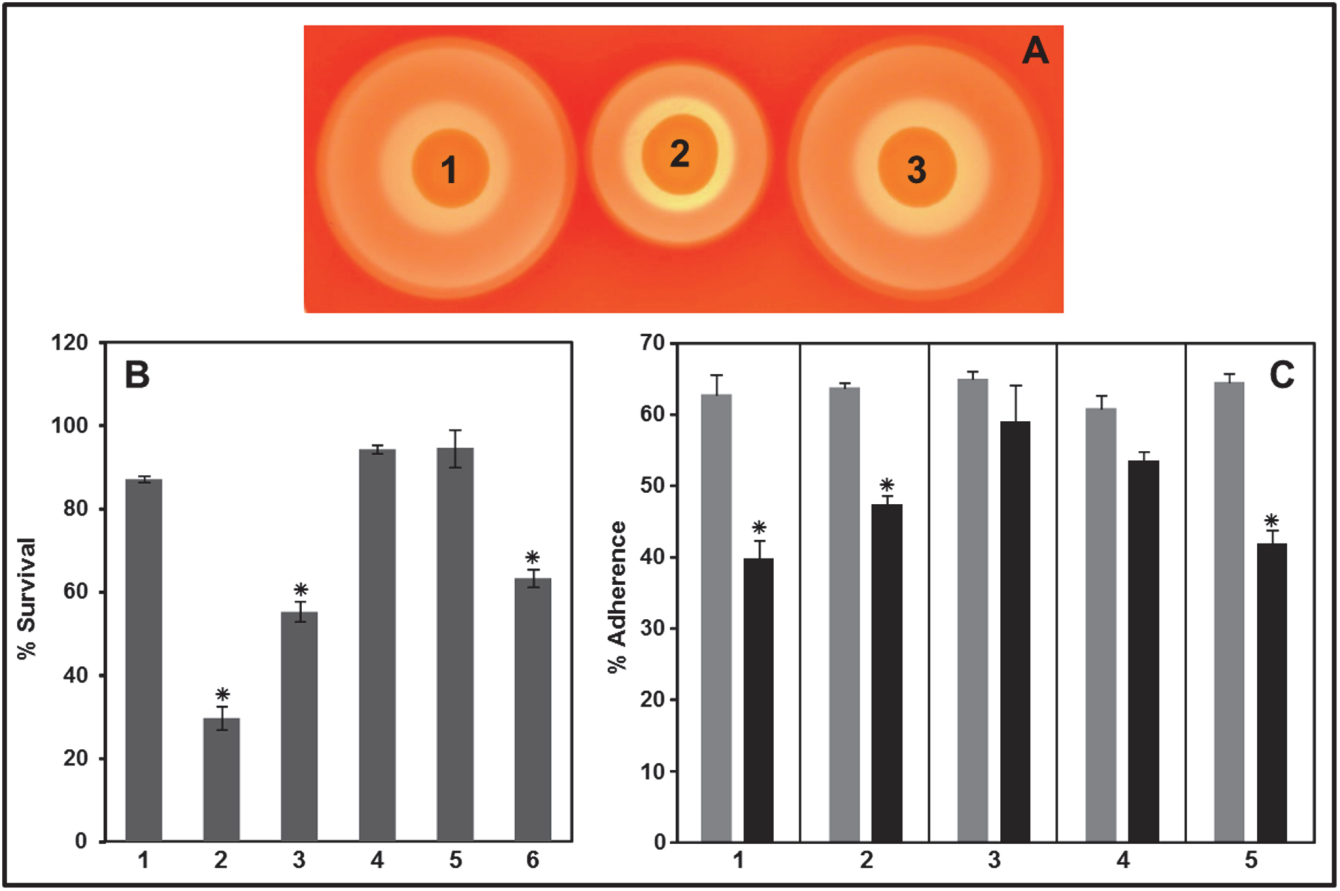
Hemolysis (A), phagocytic survival (B) and adherence (C) of complemented triple and quadruple mutants. A. Hemolysis on 5% sheep blood agar plates. (1) SH1000+pCU1, (2) SH1000:*msrA*+pCU1, (3) SH1000:*msrA*+*msrA1*. B. PMN cells were infected (MOI 1:2.5) with wild-type, mutants, and the complemented strains for 1 h at 37˚C and then plated for enumeration. (1) SH1000+pCU1, (2) SH1000:*msrA*+pCU1, (3) SH1000:*msrAB*+pCU1, (4) SH1000:*msrA*+*msrA1*, (5) SH1000:*msrAB*+*msrA1*, (6) SH1000:*msrAB*+*msrB*. Values indicate the average of three independent experiments ± standard deviation (* significant at *p*≤.05). C. The left light bar in each panel represents the ratios of the mutant or the complemented strain relative to SH1000-pCU1 in the mixture used to infect the A549 cells. The right dark bar in each panel represents the ratios of the mutant or the complemented strain in the mixture that adhered after 1 h of incubation. (1) SH1000:*msrA*+pCU1, (2) SH1000:*msrAB*+pCU1, (3) SH1000:*msrA*+*msrA1*, (4) SH1000:*msrAB*+*msrA1*, (5) SH1000:*msrAB*+*msrB*. Values indicate the average of three independent experiments ± standard deviation (* significant at *p*≤.05).

The triple SH1000:*msrA* mutant showed a defective pattern in hemolysis but its complementation with the *msrA1* gene *in trans* was shown to restore the level of hemolysis shown with the wild-type SH1000 (Fig. 9A, Spot 3). In phagocytic killing assays, the triple SH1000:*msrA* and the quadruple SH1000:*msrAB* mutants were more sensitive than the wild-type SH1000. In complementation experiments, when triple and quadruple mutants were complemented with the *msrA1* gene *in trans*, these strains showed phagocytic resistance that was comparable to wild-type SH1000 (Fig. 9B). However, complementation of the quadruple mutant with the *msrB* gene *in trans* did not restore the phagocytic resistance in these strains (Fig. 9B). Similarly, in adherence experiments, complementation with *msrA1* gene *in trans*, restored the defect in adherence that was initially seen in case of the triple or quadruple mutants (Fig. 9C). Complementation with *msrB*, on the other hand, had no appreciable effect on the adherence of the quadruple effect (Fig. 9C).

### Survival of *msr* mutants in mice

**Fig 10:**
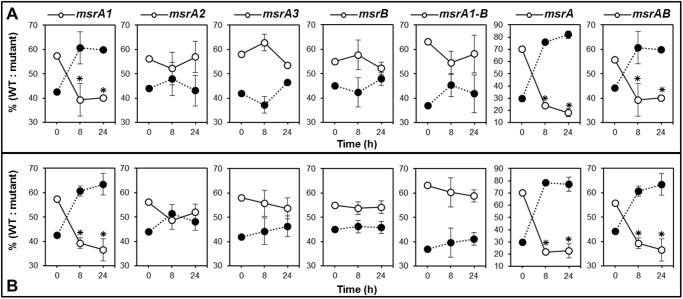
Survival of the wild-type *S*. *aureus* strain SH1000 and its derivative *msr* mutant cells in mouse. Approximately 1.0X10^8^ CFUs (predominantly mutant cells) were injected intra-peritoneally into mice. Three mice were sacrificed at 8 and 24 h post-injection. Closed circles represent the fraction of wild-type SH1000 and the open circles represent the *msr* mutant bacteria recovered from murine spleens (A) and murine livers (B) at 8 and 24 h post infection. Values at 0 h indicate the fraction of *msr* mutants and isogenic wild-type cells in the injected inoculum. Values indicate the average of three independent experiments ± standard deviation (* significant at *p*≤.05).

To elucidate the role of Msr in virulence of *S*. *aureus*, Swiss white female mice were injected with a bacterial mixture of wild-type *S*. *aureus* SH1000 and its derivative seven *msr* mutants (40:60 ratio of wild-type to mutant). The data suggest that the *msrA1* mutant of *S*. *aureus* had a lower survival rate in mice. Post infection, the fraction of *msrA1* mutants in spleen and liver was lower at 8 h and declined even further at 24 h in these tissues relative to their fraction in the mixture that was injected into the mice (Fig. 10). Loss of MsrA2, MsrA3, or MsrB had little to no effect on the survival of *S*. *aureus* in mice (Fig. 10). The triple *msrA* mutant showed the highest decline in spleen and liver tissues with time suggesting some roles for MsrA2 and MsrA3 under MsrA1-deficient conditions (Fig. 10). Although, there is a slight growth defect in the *msrA1* and triple *msrA* mutants as shown in Fig. 2, when cultured at 37°C *in vitro*, it is highly unlikely that there was much of a growth of the wild-type or the mutant bacteria in mice during our experiments that lasted only 24 h. Most of the bacteria that were injected were cleared in mice with time, as we recovered fewer bacteria after 8 h and far fewer bacteria after 24 h. It is indeed the lack of MsrA1 that significantly reduced the survival of *S*. *aureus* in mice.

### Localization of *msr* protein

**Fig 11:**
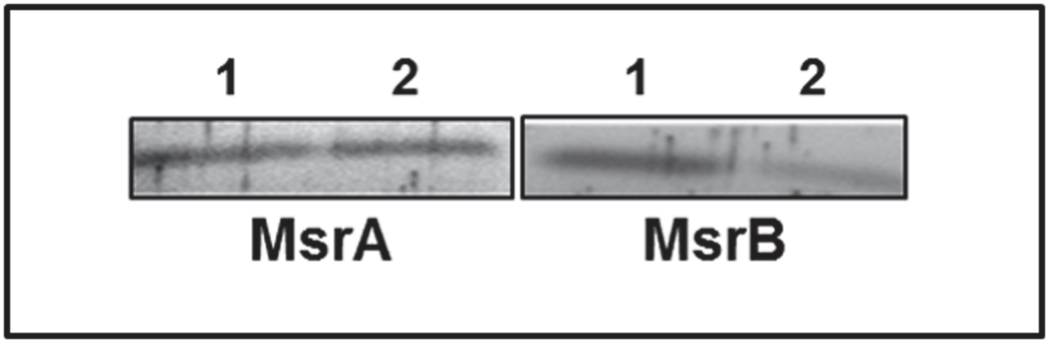
Distribution of MsrA1 and MsrB in the cytosolic and membrane fractions in *S*. *aureus* SH1000. Lane 1 indicates the cytosolic fraction and Lane 2 indicates the membrane fraction.

Localization was only investigated for MsrA1 and MsrB proteins because these two proteins have been shown to be expressed in *S*. *aureus* at a significantly higher level relative to MsrA2 and MsrA3 in *S*. *aureus* [20]. In addition, findings of this study suggest that the lack of MsrA1 or MsrB has a pleiotropic effect on *S*. *aureus* cells. Experiments utilizing anti-MsrA1 and anti-MsrB rabbit polyclonal antibodies demonstrated that the MsrA1 protein is distributed equally between the cytosolic and the membrane components in *S*. *aureus* (Fig. 11, Lanes 1 and 2). However, the MsrB protein appears to be predominantly a cytosolic protein and only a minor fraction of this protein is targeted into the bacterial membrane (Fig. 11, Lanes 3 and 4).

## Discussion

Numerous investigations in recent years have led to an increased interest and understanding of the biology of the methionine sulfoxide reductases. The reasons underlying this interest are because of a remarkable conservation across prokaryotes and eukaryotes, the importance in oxidative stress, and the novel protein repair functions of these enzymes. The two distinct Msr proteins, MsrA and MsrB, share no sequence homology. Orthologs of *msrA* and *msrB* show great variation in their genetic organization in bacterial chromosomes. In some bacterial species, the genes encoding MsrA and MsrB are located adjacent to each other and co-transcribed, and in others, the *msrA* and *msrB* genes are transcriptionally fused [17,39]. In addition, many bacterial species have multiple copies of these *msrA* and *msrB* genes distributed randomly in the bacterial chromosome and some are present even on plasmids [39].

*S*. *aureus* produces three different MsrA proteins (MsrA1, MsrA2 and MsrA3) and one MsrB protein. MsrA1 and MsrB production in *S*. *aureus* are induced by cell wall-active antibiotics. In the presence of these antibiotics, the cell wall is likely destabilized and the oxidizing agents have easy access to bacterial membrane and cytosolic compartments. In response, the staphylococcal cells produce a higher level of MsrA1 and MsrB; however, oxidative stress has not been shown to induce the synthesis of these proteins in *S*. *aureus*. In addition to these four Msr proteins, there is an additional gene (*fRMsr*) in *S*. *aureus* (SACOL1768 in *S*. *aureus* strain COL) that codes for a protein that reduces the free methionine sulfoxide. Although the structural and biochemical properties of this protein have been determined [40], its physiological relevance is unclear. The extent of expression of *S*. *aureus fRMsr* is also not clear. The *fRMsr* gene in *S*. *aureus* may be expressed at a very low level since there was no detectable Msr activity in the *msrAB* quadruple mutant (Table 3).

Studies with the individual *msr* gene mutants make it clear that the MsrA2 and MsrA3 contribute little to cellular Msr activities, play a little to no role in protecting *S*. *aureus* from oxidative stress and neutrophils, and have no impact on bacterial survival in mice. Using promoter fusion experiments, we have previously shown that *msrA2* and *msrA3* are expressed at significantly lower levels compared to the expression of the *msrA1*-*msrB* locus in *S*. *aureus* [20]. We also measured the relative transcript levels of the *msrA2* and *msrA3* relative to the transcript level of *msrA1* in *S*. *aureus*. The expression level of *msrA2* was 8–10 log lower compared to *msrA1*, and the *msrA3*-specific transcript was almost absent in a qRT-PCR assay (data not shown). In a previous study, with promoter reshuffling, we showed that the MsrA2 protein was as effective as MsrA1 in protection from oxidative stress when its expression level was raised in *S*. *aureus* [18]. The *msrA3* gene may also be under the influence of a weaker promoter compared to the strength of the promoter that drives the transcription of the *msrA1*-*msrB* genes in *S*. *aureus*.

In contrast to MsrA2 and MsrA3, lack of either MsrA1 or MsrB showed pleiotropic effects in *S*. *aureus*. The lack of MsrA1 increased the sensitivity of *S*. *aureus* to oxidative stress. Studies with a triple *msrA* mutant, which lacked all three MsrA proteins and therefore had no apparent capability to reduce *S*-MetO, showed a further increase in bacterial sensitivity to oxidants compared to only MsrA1-deficient *S*. *aureus*. This phenomenon suggests that, even though MsrA2 and MsrA3 are present at very low levels in *S*. *aureus*, they may be somewhat relevant in protecting *S*. *aureus* under MsrA1-deficient conditions. The triple *msrA* mutant also showed reduced hemolysis and increased susceptibility to neutrophil-mediated killing. This observation was expected given that MsrA deficiency in several organisms leads to enhanced vulnerability to oxidative stress [18,41,42,43]. In addition, the *msrA1* gene was up-regulated in neutrophils [12]. Within the neutrophils, the staphylococcal two-component regulatory system VraSR contributes to the *msrA1* up-regulation [12].

The MsrA1-deficient strains showed reduced pigmentation compared to the wild-type *S*. *aureus*. It has been previously shown that the staphyloxanthin pigment plays an important role in the protection of *S*. *aureus* from oxidants and neutrophils and regulates bacterial membrane fluidity and virulence [44,45,46,47]. In this study, the MsrB-deficient *S*. *aureus* strains were more pigmented and more resistant to H_2_O_2_ and cell wall-active antibiotics. One possible explanation for this phenomenon is that an increased pigmentation in the MsrB-deficient *S*. *aureus* may contribute to an impermeable membrane that restricts the oxidants and antibiotics. In turn, this change may minimize damage to cellular components under these adverse conditions. We also noted that the MsrA1-deficient *S*. *aureus* or *S*. *aureus* that was deficient in all three MsrA proteins were less adherent to human lung epithelial cells and showed reduced survival in mouse spleen and liver. The quadruple *msrAB* mutant of *S*. *aureus* also showed reduced adherence to A549 cells and survival in mouse tissues. Furthermore, the complementation experiments with the triple and quadruple mutants provide evidence that it is the MsrA1 not MsrB that is critical for staphylococcal adherence to eukaryotic cells and its resistance to the killing by phagocytic cells.

With respect to the role of the Msr proteins, it is well documented that these enzymes contribute to the ability of a pathogen to adhere to host tissue, evade immune system, form biofilms, survive inside macrophages, and resist oxidative killing [14].MsrA protein contributes to cell wall integrity and maintenance of adhesion properties in *Streptococcus gordonii* [48]. Msr proteins have also been shown to affect adherence properties of pathogenic *Neisseria* [17]. In *S*. *gordonii*, the MsrA enzyme was shown to maintain the integrity of bacterial adhesins during oxidative stress [49]. The current study confirms the role of Msr proteins, particularly the MsrAs in the adherence of *S*. *aureus* to human cells. The MsrA1-deficient *S*. *aureus*, the triple *msrA* and the quadruple *msrAB* null-mutants, all showed reduced adherence to lung epithelial cells. The role of Msr proteins in virulence of the bacterial pathogens is also well documented. Both MsrA and MsrB contributed to the enzymatic defenses of *Mycobacterium tuberculosis* from reactive oxygen species [50]. In *Pseudomonas aeruginosa*, inactivation of either *msrA* or *msrB* or both reduced virulence and increased its killing by oxidants [51]. In *Campylobacter jejuni*, the single *msrA* or *msrB* mutants showed no growth defect, but the *msrA*-*msrB* double mutant showed increased sensitivity to oxidative stress conditions [31]. Mutation in the *msrA* or *msrB* gene in *Enterococcus faecalis* resulted in increased sensitivity to H_2_O_2_. In addition, an *msrA msrB* double mutant showed further increase in sensitivity suggesting that the effect of mutations were additive [15]. In a later study, however, the *msrA* and *msrB* mutants were shown to behave differently; the *msrA* mutant was more sensitive to oxidative stress conditions whereas the *msrB* mutant showed stimulated growth under similar conditions [52]. In *Salmonella* Typhimurium, deletion of *msrA* increased bacterial susceptibility to H_2_O_2_ and reduced its virulence, but a mutation in *msrB* had no apparent phenotype [11]. In *Mycobacterium smegmatis* also, MsrB was shown to have a limited role in protection from oxidative stress conditions [53].

Thus, the role of MsrB protein in defense from oxidative stress is questionable in many bacterial species. It is possible that under oxidative stress the majority of the oxidized methionine is *S*-MetO and the MsrB protein has no activity against this epimer. This may be the reason why the MsrA-deficient bacteria showed a high sensitivity to conditions that impose oxidative stress. MsrB of *S*. *aureus*, seems to some extent, counterbalance the effect of MsrA1. For example, lack of MsrA1 reduces pigmentation and this may be due to previously shown higher level of MsrB in MsrA1-deficient *S*. *aureus* [18]. However, when MsrB is absent, the bacterium responds by increasing pigment production as a potential compensatory mechanism.

In summary, among the four Msr enzymes produced in *S*. *aureus*, MsrA2 and MsrA3 contribute little to the enzymatic activity and bacterial defense from oxidative stress. MsrA1 and MsrB have opposing roles in pigment production and resistance from oxidative stress. MsrA1 seems to be equally distributed between the cytosolic and membrane components but the MsrB appears to be predominantly cytosolic. Regulation of *msrA1*-*msrB* locus is currently under investigation because of its significant role in *S*. *aureus* physiology and virulence.
